# A Complication-Based Comparison Between the Posterior and Direct Lateral Approaches to Total Hip Arthroplasty: A Single-Center Experience

**DOI:** 10.7759/cureus.12469

**Published:** 2021-01-04

**Authors:** Wazzan ALJuhani, Khalid Alshuwaier, Fisal Alkhamis, Mohammed Q Alosaimi, Abdullah Alaidroos, Mohammad A Alghafees, Emad Masuadi

**Affiliations:** 1 Surgery, King Abdulaziz Medical City, Riyadh, SAU; 2 Medicine, King Saud Bin Abdulaziz University for Health Sciences, Riyadh, SAU; 3 Research Unit/Biostatistics, King Saud Bin Abdulaziz University for Health Sciences, Riyadh, SAU

**Keywords:** arthroplasty, complications, orthopaedic surgery, surgical approaches, saudi arabia

## Abstract

Introduction

Many approaches to performing total hip arthroplasty (THA) exist, primarily due to an insufficient amount of research that would favor one approach over the other. This study aimed to compare the risk of nerve injury, dislocation, Trendelenburg gait, and stem malposition between the direct lateral and posterior approaches to THA.

Methods

The study was a retrospective cohort study, and it was conducted in King Abdulaziz Medical City. It was directed toward adult patients who underwent THA from November 1, 2003, to November 1, 2018. All figures were obtained through the BESTCare system (ezCareTech, Saudi Arabia). Categorical variables were presented as frequencies and proportions. Quantitative variables were measured as mean and standard deviation. Fisher's exact test was used to compare the risk of complications between the two approaches.

Results

The posterior approach displayed a greater risk of stem malposition (p< 0.001) when compared with the direct lateral approach. However, neither approach showed a higher risk of dislocation, nerve injury, or Trendelenburg gait.

Conclusions

A higher risk of stem malposition was observed in the posterior approach, and there were no remarkable variances between the two approaches in the risk of dislocation, nerve injury, and Trendelenburg gait. Until more meticulous nationwide studies are available to provide evidence that would favor an approach over the other, the choice of surgical approach for THA remains to depend on the patient's characteristics and the surgeon's prior experience. Regardless of the approach, preoperative, intraoperative, and postoperative factors that increase the risk of complications should also be identified and addressed adequately.

## Introduction

Total hip arthroplasty (THA) is one of the most common procedures in the world. For instance, in 2010, total hip replacement occurrence in the United States was less than 1% of all arthroplasty surgeries. The estimation was 2.5 million individuals divided into 1.4 million females and 1.1 million males [1].

There is a plethora of approaches to performing total hip arthroplasty. A surgeon can use any approach depending on their own personal preference; because, regarding safety, no adequate amount of convincing research would favor an approach over the other. The posterior and direct lateral approaches are currently two of the most widely used approaches [2].

This study aimed to compare the risk of nerve injury, dislocation, Trendelenburg gait, and stem malposition between both the direct lateral and posterior approaches to the THA procedure in King Abdulaziz Medical City. This research has substantial significance due to the lack of similar studies in the region, i.e., the Middle East.

## Materials and methods

The study design is a retrospective cohort and conducted at King Abdulaziz Medical City, Riyadh, Saudi Arabia (KAMC). Were incorporated in the study, adult patients who underwent THA in the posterior or direct lateral approaches at KAMC with full medical records from November 1, 2003, to November 1, 2018. Patients who underwent THA outside of KAMC but were being followed up in the aforementioned hospital or underwent THA in an approach other than the posterior or direct lateral were excluded from the study.

The BESTCare system (ezCareTech, Saudi Arabia) was used to collect data. The study included all adult patients who undertook THA in the specified time period except those who have not matched the inclusion criteria. The main resulted variables were nerve injury, dislocation, Trendelenburg gait, and stem malposition. Patients' information was used to group the variables, including age, gender, body mass index (BMI), comorbidities, indications, and admission type.

Figures were conducted by Microsoft Excel 2019 (Microsoft Corporation, WA, USA) and were studied using the Statistical Package for the Social Sciences (SPSS) version 23.0 (IBM Corporation, NY, USA). Frequencies and proportions were brought about for categorical variables, whereas mean and standard deviation were computed for numerical variables. To make a comparison of the risk of complications between the two approaches, Fisher's exact test was used. A p-value of ≤ 0.05 was deemed significant.

The Institutional Review Board of King Abdullah International Medical Research Center, the Ministry of National Guard Health Affairs, Riyadh, Kingdom of Saudi Arabia, approved the present study with approval number SP19/369/R. Patients' confidentiality was ensured. Only the research team members used and collected the data and serial numbers were used instead of medical record numbers.

## Results

Of the 126 participants, 64.3% (n = 81) were males and 35.7% (n = 45) were females. The mean age was 49.32 ± 19.56 years. The mean body mass index (BMI) was 29.14 ± 6.44 kg/m2, as shown in Table 1.

**Table 1:** The Demographic Profile and Comorbidities of The Study Participants (N = 126)

Demographical Data		
Age (Mean, SD)	49.32	19.56
Male (N, %)	81	64.30
Female (N, %)	45	35.70
Body Mass Index (Mean, SD)	29.14	6.44
Comorbidities	N	%
Sickle Cell Disease	8	6.30
Dyslipidemia	26	20.60
Hypertension	41	32.50
Diabetes Mellitus	17	13.50
Hypothyroidism	8	6.30
Hip Dysplasia	6	4.80
Hepatitis C	1	0.80
Renal Failure	4	3.20
Osteoporosis	7	5.60
Deep Vein Thrombosis	4	3.20
Benign Prostatic Hyperplasia	9	7.10
Congestive Heart Failure	12	9.50
Arrhythmias	7	5.60
Chronic Obstructive Pulmonary Disease	7	5.60
Rheumatoid Arthritis	1	0.80
Spondylitis	3	2.40
Osteoarthritis	5	4.00
Avascular Necrosis	3	2.40
Thalassemia	3	2.40

Of the comorbidities, the most common was hypertension (32.5% [n = 41]) followed by dyslipidaemia (20.6% [n = 26]), diabetes mellitus (13.5% [n = 17]), and congestive heart failure (9.5% [n = 12]), as shown in Table 1.

Of the 126 participants, 77% (n = 97) were admitted for THA electively, and 23% (n = 29) were admitted urgently. Among the different indications, avascular necrosis was the most common (36.5% [n = 46]) followed by post-traumatic osteoarthritis (19.8% [n = 25]), arthritis (17.5% [n = 22]) and fractures (13.5% [n = 17]). The direct lateral approach of THA was done on 86.5% (n = 109) while 13.5% (n = 17) underwent THA in the posterior approach. The most common surgical complication observed was dislocation (11.9% [n = 15]), followed by nerve injury (8.7% [n = 11]), stem malposition (6.3% [n = 8]), and Trendelenburg gait (6.3% [n = 8]), as shown in Table 2.

**Table 2:** Operation Details and Complications (N = 126)

Admission Type	N	%
Elective	97	77
Urgent	29	23
Indication	N	%
Avascular Necrosis	46	36.5
Fractures	17	13.5
Rheumatic Diseases	1	0.8
Arthritis	22	17.5
Post-traumatic Osteoarthritis	25	19.8
Dysplasia	15	11.9
Surgical Approach	N	%
Posterior Approach	17	13.50
Direct Lateral Approach	109	86.5
Complication	N	%
Trendelenburg Gait	8	6.3
Nerve Injury	11	8.7
Dislocation	15	11.9
Stem Malposition	8	6.3

A substantial difference was detected between the two approaches regarding having stem malposition, where a higher risk of stem malposition was observed in the posterior approach (p < 0.001). No significant differences were observed between the two approaches as per the risks of dislocation, nerve injury, and Trendelenburg gait, as shown in Table 3.

**Table 3:** Surgical Approach-Based Comparison of Complications

Complication	Type of Surgical Approach				P-Value
	Posterior Approach (N =17)		Direct Lateral Approach (N = 109)		
Trendelenburg Gait	N	%	N	%	0.248
Yes	0	0.00	8	7.34	
No	17	100.00	101	92.66	
Nerve Injury	N	%	N	%	0.655
Yes	1	94.10	10	90.80	
No	16	5.90	99	9.20	
Dislocation	N	%	N	%	0.103
Yes	0	0.00	15	13.80	
No	17	100.00	94	86.20	
Stem Malposition	N	%	N	%	< 0.001*
Yes	7	41.20	1	0.90	
No	10	58.80	108	99.10	

## Discussion

The study aimed at comparing the risks of nerve injury, dislocation, Trendelenburg gait, and stem malposition between the direct lateral and posterior approaches to total hip arthroplasty in KAMC. We observed a higher risk of stem malposition in the posterior approach than the direct lateral approach. However, the study found no principal variances between the two approaches as per the risks of dislocation, nerve injury, and Trendelenburg gait.

The respective superiority of a THA approach over the other is a controversial subject, with conflicting published results. Unlike a British meta-analysis, which found the posterior approach to have a lower risk of stem malposition, the present study found that the posterior approach had a higher chance of stem malposition than the lateral approach. The study also found the posterior approach to have a lower risk of Trendelenburg gait, which also contradicts our findings. However, the study did not find any difference regarding dislocation risk between the two approaches, which is consistent with the presented findings [3]. Moreover, unlike a systemic review that found a higher risk of nerve injury in the direct lateral approach, our study found no significant difference between the two approaches. However, akin to our negative findings, the same study found that none of the two approaches showed a higher risk of dislocation and postoperative Trendelenburg gait [4]. Moreover, dissimilar to a Swedish study that uncovered a larger chance of dislocation when using the posterior approach, the current study found no difference in dislocation peril between the posterior and direct lateral approaches [5].

Furthermore, a prospective American study found no statistically significant difference between the posterior and lateral approaches in nerve palsy risk, which complements our study's negative findings [6]. On the contrary, a high-potential Brazilian study found an association between using the direct lateral approach and the risk of damage to the superior gluteal nerve, which contradicts our findings [7]. A meta-analysis assessing the risk of dislocation in each of the three most common approaches, including the posterior and the direct lateral approach, found that the dislocation rates are similar, further complementing our negative findings [8]. Furthermore, an additional meta-analysis did not find any association between a greater peril of dislocation and the posterior approach [9]. Inversely, a paper reviewing 260 clinical studies specified that from the data collected, the dislocation rate following THA was 5.9 times higher with the posterior approach compared to the direct lateral approach, which in turn conflicts with our negative findings [10]. Also, a Swiss case-control study found that patients undergoing the posterior approach were six times more prone to experiencing dislocation than the direct lateral approach [11]. In contrast, a South Korean study found that patients are less prone to developing a dislocation when undergoing THA in the posterior approach than in the lateral approach [12]. Moreover, a Dutch study found that following governing relevant perplexing variables, the lateral approach to THA presented a higher risk of stem malposition when compared with the posterior approach, which contradicts our findings [13].

Regardless of the approach, these complications can be prevented through preoperative and intraoperative measures. According to a Spanish cohort study, the capsular repair and transosseous reattachment of the external rotators can excellently decrease the peril of dislocation in patients undergoing THA in the posterior approach [14]. Also, a French multicentre case-control study found that an unrepaired joint capsule is considered a significant risk factor for dislocation in patients undergoing THA in any approach. However, the risk was highest in the group that underwent THA in the posterior approach [15]. Also, a British study found that age extremes, a BMI >30 kg/m^2^, lumbosacral disease, and femoral head size can all influence the rates of dislocation post THA irrespective of the approach used [16]. Furthermore, a German study stated that the observed critical trochanter angle in preoperative planning could help in predicting the risk for stem malpositioning. The study recommended that an intraoperative C-ray be used to verify the correct positioning of an implant in patients with a critical trochanteric angle under 20° or above 30° [17].

There were some limitations to the study. First, the population size was relatively smaller than global, multicentre studies. Second, due to the study's retrospective nature, the data in the BESTCare system might not be complete. Concerning strengths, first, the current study is the first of its kind in the Middle East and would be a good initiative for conducting more extensive studies that include nationwide databases. Second, King Abdulaziz Medical City follows similar prehabilitation and rehabilitation protocols for all patients undergoing THA, which further solidifies the evidence of approach-related complications.

## Conclusions

A higher risk of stem malposition was observed in the posterior approach, and no significant differences were found between the two approaches in the risk of dislocation, nerve injury, and Trendelenburg gait. Until more meticulous nationwide studies are available to provide strong evidence that would favor one approach over the other, the choice of surgical approach for THA remains dependent on the patient's characteristics and the surgeon's experience. Due to the lack of studies, we recommend that more clinical trials and more extensive nationwide database cohort studies be conducted to explore more factors that would give an advantage to one approach over the other. Future research should also focus on the long-term impact of different surgical approaches on the patient's prognosis. Regardless of the approach, the preoperative, intraoperative, and postoperative factors that increase the risk of complications should also be identified and addressed.
